# Identification of Quantitative Trait Loci and Water Environmental Interactions for Developmental Behaviors of Leaf Greenness in Wheat

**DOI:** 10.3389/fpls.2016.00273

**Published:** 2016-03-08

**Authors:** Delong Yang, Mengfei Li, Yuan Liu, Lei Chang, Hongbo Cheng, Jingjing Chen, Shouxi Chai

**Affiliations:** ^1^Gansu Provincial Key Lab of Aridland Crop Science/School of Life Science and Technology, Gansu Agricultural UniversityLanzhou, China; ^2^School of Agronomy, Gansu Agricultural UniversityLanzhou, China

**Keywords:** *Triticum aestivum*, leaf greenness, drought stress, developmental genetics, QTL mapping, water environmental interactions

## Abstract

The maintenance of leaf greenness in wheat, highly responsible for yield potential and resistance to drought stress, has been proved to be quantitatively inherited and susceptible to interact with environments by traditional genetic analysis. In order to further dissect the developmental genetic behaviors of flag leaf greenness under terminal drought, unconditional and conditional QTL mapping strategies were performed with a mixed linear model in 120 F_8_-derived recombinant inbred lines (RILs) from two Chinese common wheat cultivars (Longjian 19 × Q9086) in different water environments. A total of 65 additive QTLs (A-QTLs) and 42 pairs of epistatic QTLs (AA-QTLs) were identified as distribution on almost all 21 chromosomes except 5A, explaining from 0.24 to 3.29 % of the phenotypic variation. Of these, 22 A-QTLs and 25 pairs of AA-QTLs were common in two sets of mapping methods but the others differed. These putative QTLs were essentially characteristic of time- and environmentally-dependent expression patterns. Indeed some loci were expressed at two or more stages, while no single QTL was continually active through whole measuring duration. More loci were detected in early growth periods but most of QTL × water environment interactions (QEIs) happened in mid-anaphase, where drought stress was more conducted with negative regulation on QTL expressions. Compared to other genetic components, epistatic effects and additive QEIs effects could be predominant in regulating phenotypic variations during the ontogeny of leaf greenness. Several QTL cluster regions were suggestive of tight linkage or expression pleiotropy in the inheritance of these traits. Some reproducibly-expressed QTLs or common loci consistent with previously detected would be useful to the genetic improvement of staygreen types in wheat through MAS, especially in water-deficit environments.

## Introduction

Wheat (*Triticum aestivum* L.) is one of the most important foodstuff crops in semiarid and arid areas around the world. As current changes in global climate have increased the precipitation variability with frequent episodes of drought (Trenberth, 2011), the wheat production in rainfed regions is strongly constrained by erratic drought stresses (Gregersen et al., 2013). In particular, terminal drought occurring during the reproductive phase in wheat is responsible for poor grain set and development, which finally results in substantial reductions in grain yield (Nawaz et al., 2013). Although, principal explanations for these losses are still complicated, it is critically associated with the drought-induced premature senescence of flag leaf (Verma et al., 2004). Here terminal drought is considered to essentially accelerate leaf chlorophyll degradation and thus impede carbon fixation (Guóth et al., 2009) and assimilate remobilization (Gregersen et al., 2008). In this context, wheat genotypes with a functional staygreen characteristic, i.e., delayed leaf senescence, can maintain photosynthetic capacity and favorable supply of assimilates to grain for a longer duration of time to assure better grain yield (Gong et al., 2005; Christopher et al., 2008; Chen et al., 2010). Therefore, the staygreen attribute of flag leaf under terminal drought is of great importance for determining wheat yield potential and resistance to drought stress (Biswal and Kohli, 2013; Farooq et al., 2014).

To develop the staygreen trait of flag leaf as an effective selection criterion in drought-tolerant breeding in wheat, much effort has been exerted to understand the genetic mechanism of the trait in wheat (Kumar et al., 2010; Naruoka et al., 2012; Barakat et al., 2013) and other cereal crops (Yoo et al., 2007; Kassahun et al., 2010; Wang et al., 2012; Emebiri, 2013). Current genetic gains in this phenotype, as reviewed by Thomas and Ougham (2014), are proved to be polygenic with functional genes and transcription factors by typical approaches of mutation, gene expression profiles and transgenic plants. Alternatively, polygenes with quantitative effects can also be developed by quantitative trait loci (QTLs) analysis (Verma et al., 2004; Yoo et al., 2007; Kassahun et al., 2010; Kumar et al., 2010; Wang et al., 2012; Emebiri, 2013). In wheat, these putative QTLs were almost mapped on all 21 chromosomes, with a widely flexible expression in response to various genetic populations and environments (Verma et al., 2004; Yang et al., 2007; Zhang et al., 2009a,b, 2010; Kumar et al., 2010; Li H. et al., 2012; Naruoka et al., 2012; Barakat et al., 2013; Czyczyło-Mysza et al., 2013). The genetic components estimated from segregation progenies of wheat crosses elucidated that the leaf staygreen trait was governed by only a few of major genes /QTLs with high predominance of additive effects (Silva et al., 2000; Verma et al., 2004; Joshi et al., 2007; Kumar et al., 2010). However, most of present studies indicated that the phenotype was under polygenes control by minor additive effects, which were variable across environments (Li H. et al., 2012; Naruoka et al., 2012; Barakat et al., 2013; Czyczyło-Mysza et al., 2013). In some cases, epistatic effects (Zhang et al., 2009a,b, 2010; Kumar et al., 2012) or QTL × environment interaction (QEI) effects (Yang et al., 2007; Peleg et al., 2009) were also highlighted in the modulation of its genetic variation. Under these observations, although flag leaf staygreen in wheat was confirmed to be inherited quantitatively, few studies have been undertaken to adequately dissect its genetic components and QEI variability in a same experiment system, especially under the terminal drought.

On the other hand, leaf staygreen *per se* is a complex developmental process (Thomas and Ougham, 2014). The statistical analysis showed that the development of such a quantitative trait occurs through the actions and interactions of polygenes and their environmental interactions, behaving differentially during different growth stages (Atchley and Zhu, 1997). Nevertheless, present QTL information for leaf staygreen in wheat was only achieved at a specific time point without considering sequential effects due to distinct gene expression at different developmental stages. Actually, it is inadequate to discover the real genetic information on the developmental processes of target quantitative traits (Wang et al., 2010). To dissect the dynamics of QTL expression, unconditional and conditional methods have been proposed (Zhu, 1995; Atchley and Zhu, 1997; Wu et al., 1999). Unconditional analysis is a traditional method for studying developmental behavior, which unravels genetic cumulative effects from the original to time *t*, rather than the real effects of gene expression during the ontogeny. Conditional analysis is another method to assess net genetic effects in the period from time (*t-*1) to time *t* on trait development. Being independent of the causal genetic effects and susceptible to the developmental status and environments, conditional analysis is valid to identify dynamic gene expression and new genetic variation arising in specific development periods (Cao et al., 2001; Wu et al., 2010). Present studies have also been verified that conditional effects in early growth periods, as normally cumulative components, could affect later unconditional effects (Wang et al., 2010; Wu et al., 2010; Li S. et al., 2012). Thus, the combination of unconditional and conditional analyses is employed to identify dynamic expressions of developmental QTLs and reveal the comprehensive inheritance of quantitative traits (Wu et al., 2010; Li S. et al., 2012). The strategy has been applied to understanding the genetic basis of crop developmental traits, such as plant height (Wang et al., 2010; Wu et al., 2010), tiller number (Yang et al., 2006; Liu et al., 2010), grain weight (Han et al., 2012; Li S. et al., 2012), grain filling (Takai et al., 2005), and seed quality (Han et al., 2011). All the studies show that the genetic architecture of developmental traits gets more involved in a time-dependent expression of polygenes through additive, epistatic and QEI effects. However, few reports so far on QTLs analysis by this methodology have documented the staygreen of flag leaf in wheat under the terminal drought stress across diverse water environments.

In this study, we used recombinant inbred lines (RILs) in wheat grown under different water environments to explore genetically the developmental behaviors of flag leaf staygreen. Using both the unconditional and conditional genetic models analyzed the trait performances at multiple observation times in the reproductive duration, respectively. The objectives of the work reported here were (1) to identify the QTLs with genetic main effects and QEI effects controlling flag leaf staygreen and (2) to unravel its dynamics of QTL expression during ontogeny, even under terminal drought stress. The findings might be valuable for well-understanding the genetic basis governing leaf staygreen development, and for wheat genetic improvement of drought tolerance by marker-assisted selection (MAS).

## Materials and methods

### Plant materials

A subset of 120 F_8_-derived RILs was developed from the cross between two Chinese winter wheat varieties, Longjian 19 and Q9086. The male parent Longjian 19 is an elite drought-tolerance cultivar widely grown in rainfed areas (300~500-mm annual rainfall) in northwestern China and was released by Gansu Academy of Agricultural Sciences, Lanzhou, China. The female Q9086 is a high-yielding cultivar alternative to relatively sufficient water and fertile conditions, but easily susceptible to pre-senescence under terminal drought stress, and was released by Northwest Agriculture & Forestry University, Yangling, China. In addition, the two parents differed significantly in major agronomical and physiological characteristics under terminal drought stress, as described in our previous studies (Yang et al., 2012, 2014; Li et al., 2013; Ma et al., 2014; Hu et al., 2015; Ye et al., 2015).

### Field trials

Two parents together with the RILs were grown at Lanzhou (103°51′ E, 36°04′ N, 1520 m Altitude), Gansu, China, in 2012–2014, denoted in turn as E1, E2, and E3, respectively. The experimental fields in each year were treated under drought stress (DS) and well-watered (WW) conditions. The DS treatment was equivalent to the rainfed condition with a total of 99.6, 113.5, and 101.8 mm rainfall during the growing season (from early October in the sowing year to late June in harvesting year), respectively. The WW treatment was irrigated with 750 m^3^ ha^−1^ water supply at the pre-overwintering, jointing, and flowering stages, respectively. The field design of each plot consisted of randomized complete blocks with three replications. Each plot was 2 m long with six rows spaced 20 cm apart with approximately 160 plants per row. Field management was conducted following the local practice in wheat production.

Leaf greenness, a surrogate measure of leaf chlorophyll content, was monitored using a Minolta Chlorophyll Meter SPAD 502 (Konica Minolta, Japan), which has been used extensively in the accurate diagnosis of the staygreen characteristics in many crops (Borrell et al., 2001; Jiang et al., 2004; Harris et al., 2007). In this study, SPAD readings were thus made a direct assessment of the degree of leaf greenness. The flag leaves of 10 main shoots growing uniform of each RIL were tagged from the middle of each plot for the assessment of the dynamic greenness degree. SPAD readings were taken at the central point of target flag leaves at each time of measurement. At the onset of flowering, SPAD values were scored every 6 d until 24 d after flowering, which duration actually covered peak grain-filling stages. Therefore, the foregoing five measurements were designated as S1, S2, S3, S4, and S5, respectively. The trait means of 10 samples from each plot with three replications were applied to the data analysis.

### Data analysis

According to the development theory proposed by Zhu (1995), the actual SPAD data measuring at the above-mentioned stages (S1–S5) were defined as unconditional values. Using the program of QGA Station based on a mixed model (Yang et al., 2006), the conditional SPAD values were generated by converting the actual SPAD data at every two consecutive occasions, designated as S1|S0, S2|S1, S3|S2, S4|S3, and S5|S4, respectively. When phenotypic values were measuring at the first time point, the unconditional genetic effects were equivalent to those obtained from conditional analysis.

Basic statistics and Pearson phenotypic correlations between the traits were performed by SAS software (SAS Institute, 1996). The broadsense heritability (*h*${}_{\text{B}}^{2}$) of the greenness of flag leaf was estimated with the method proposed by Toker (2004). To dissect the quantitative genetic basis of the developmental behavior of the post-anthesis greenness of flag leaf, its unconditional and conditional phenotypic data at five growth stages were subjected to QTL analysis. A genetic linkage map, consisting of 524 simple sequence repeats (SSRs) marker loci mapped on 21 chromosomes, was available. The map was covered 2266.7 cM with an average distance of 4.3 cM between adjacent markers (Hu et al., 2015; Ye et al., 2015). QTL analysis was implemented by the mixed linear model mapping (Wang et al., 1999), using the Windows version computer program QTLNetwork-2.0 (Yang et al., 2008). The genetic model could divide genetic effects into additive effects (*A*), epistatic effects (*AA*), and QEI effects (including *AE* and *AAE*). An experiment-wise type I error of 0.05 was designated for candidate interval selection and putative QTL detection. The critical *F*-value to declare putative QTLs and to control genome-wise type I errors was accommodated by 1000 permutation tests. Both the testing and filtration window were set at 10 cM, with a walk speed of 2 cM. QTLs were named according to the rule of ‘QTL+trait+research department+chromosome’.

## Results

### Phenotypic variation and trait correlation

The genotypic means for SPAD values as the greenness of flag leaf in all treatments are summarized in Table 1. The phenotypic variations of both RILs and two parents showed a progressive depletion trend with the growth progression across water environments. Moreover, phenotypic values in the DS were significantly lower than those in the WW. The parents, Longjian 19 and Q9086, differed in consecutive traits in response to the water regimes. Under the WW, phenotypic values of Q9086 at most of stages were higher than those of Longjian 19, whereas the case in the DS was reverse and much noticeable. This suggested Longjian 19 was capable of stronger staygreen to withstand drought stress. Across all measuring stages, the mean values of RILs displayed a consistent reduction and were intermediate between those of the two parents. Highly phenotypic variability was found in the population, with coefficients of variation (CV) ranging from 6.31 to 27.00% in the DS and from 4.91 to 15.76% in the WW depending on different stages and water environments. Some lines had more extreme values than the parents, showing substantial transgressive segregation. All skewness and kurtosis values were less than 1.0 occurring at all treatments, suggestive of their continuous distributions and quantitative bases.

**Table 1:** **Phenotypic performance for the greenness (SPAD values) of flag leaf of the parents and RILs at five growth stages in different water environments**.

**Environ^a^**.	**Stage^b^**	**Parents**		**RILs**					
		**Longjian19**	**Q9086**	**Mean**	**Min**.	**Max**.	**CV (%)^c^**	**Skewness**	**Kurtosis**
E1	S1	46.52/50.05	41.46/54.83	45.10/53.66	34.23/42.10	52.73/54.83	7.21/5.41	0.29/−0.17	−0.22/−0.66
	S2	45.15/50.97	40.03/53.46	42.46/52.72	31.01/41.27	49.31/54.49	6.68/5.70	0.01/−0.15	−0.59/−0.62
	S3	40.50/49.72	31.68/50.14	36.46/49.95	28.20/35.50	43.00/51.17	7.16/4.91	0.07/−0.26	−0.21/0.22
	S4	32.38/44.63	15.71/45.78	23.03/44.13	18.18/29.73	33.62/47.52	14.96/6.68	−0.15/0.17	−0.15/−0.57
	S5	20.27/35.81	12.85/32.15	15.06/33.04	12.78/20.53	22.15/35.33	22.17/15.76	0.60/0.28	0.08/−0.41
E2	S1	50.74/52.16	46.55/55.95	47.32/53.73	36.61/44.42	54.98/55.65	6.31/5.24	0.33/−0.09	−0.21/−0.59
	S2	47.96/50.84	41.28/55.70	43.73/52.85	33.38/43.02	52.76/54.68	6.42/5.87	−0.15/−0.07	−0.24/−0.53
	S3	42.58/49.13	34.64/51.58	38.93/50.68	30.24/39.06	46.80/53.41	6.71/5.45	0.05/−0.34	−0.92/0.10
	S4	38.43/47.50	19.78/48.47	26.22/47.71	21.94/33.57	38.48/50.33	11.93/6.72	0.06/0.08	−0.24/−0.55
	S5	24.90/39.69	14.32/35.86	17.68/36.54	13.52/23.11	25.89/39.18	25.57/14.48	0.61/0.40	0.76/−0.11
E3	S1	46.52/51.43	42.60/54.65	44.57/52.22	33.40/42.94	53.41/55.30	6.99/5.30	0.35/−0.16	−0.10/−0.43
	S2	44.96/51.58	37.38/54.01	40.02/52.41	32.35/40.62	50.08/55.04	7.44/5.91	−0.17–0.14	−0.18/−0.38
	S3	41.85/49.72	30.77/51.93	37.19/50.76	29.55/37.58	45.09/51.80	7.60/5.54	0.05/−0.41	−0.96/0.43
	S4	33.28/41.63	17.5/42.75	24.48/41.86	21.05/31.64	36.97/48.61	16.90/7.99	0.07/0.07	−0.20/−0.37
	S5	22.43/36.81	12.25/34.15	15.36/33.25	13.15/22.27	24.47/37.53	27.00/13.80	0.55/0.41	0.71/−0.08

*The numbers at the left of the slash (“/”) are the phenotypic values of traits identified in the drought stress, and the numbers at the right indicate the well-watered condition*.

a *E1–E3 represent the location at Lanzhou (103°51′ E, 36°04′ N, 1520 m Altitude), Gansu, China, in 2012–2014, respectively*.

b *S1–S5 indicate the first to the fifth measuring stage, respectively*.

c *CV(%) means the coefficient of variation*.

Results of variation component analysis (Table 2) showed that all the variances for both unconditional and conditional values in RILs reached the 0.05 or 0.01 significant level, except for the interaction variances of both genotype × environment and genotype × environment × water. In contrast, the dominant source of variations for unconditional and conditional traits was the water regime, which accounted for 83.82–93.26% and 83.82–95.42% of total variation, respectively. This indicated that water environments could exert more considerable influence on the ontogeny of flag leaf greenness. Despite the substantial variation, the *h*${}_{\text{B}}^{2}$ estimates were reasonable for both unconditional and conditional values across environments, differing from 0.34 to 0.64 and from 0.31 to 0.64, respectively, which were present in a gradual decline trend with developmental stages.

**Table 2:** **Analyses of variance (ANOVA) for the greenness of flag leaf in RILs at five growth stages**.

**Source of variation**	***df***	**Mean square**				
		**S1/S1|S0**	**S2/S2|S1**	**S3/S3|S2**	**S4/S4|S3**	**S5/S5|S4**
Water(W)	1	16612.78^**^/16612.78^**^	30119.34^**^/4272.15^**^	62388.58^**^/2735.29^**^	124672.16^**^/4943.98^**^	69656.65^**^/3916.81^**^
Environment(E)	2	2920.48^**^/2920.48^**^	4249.51^**^/256.80^**^	2758.76^**^/234.53^**^	14966.08^**^/322.80^**^	3945.03^**^/303.87^**^
Genotype(G)	119	154.39^**^/154.39^**^	159.91^**^/19.72^**^	111.63^**^/21.63^**^	179.31^**^/54.81^**^	101.85^**^/49.26^**^
W × E	2	74.38^**^/74.38^**^	416.66^**^/19.04^**^	57.31^**^/12.49^**^	125.62^**^/67.83^**^	133.71^**^/15.13^**^
W × G	119	49.18^**^/49.18^**^	54.73^**^/7.57^*^	51.17^**^/8.31^*^	87.74^**^/26.79^**^	58.28^**^/27.85^**^
E × G	238	3.73/3.73	6.90/1.29	4.76/2.28	4.26/4.02	6.01/4.24
W × E × G	238	1.32/1.32	3.92/0.94	1.77/2.04	2.32/2.53	3.27/1.75
Error	1440	2.97/2.97	3.64/3.26	6.22/5.61	6.99/6.86	7.95/5.88
*h*${}_{\text{B}}^{2}$		0.64/0.64	0.59/0.50	0.48/0.41	0.47/0.39	0.34/0.31

*The numbers at the left of the slash (“/”) are mean squares or heritability for the unconditional phenotype of RILs, and the numbers at the right indicate the conditional phenotype of RILs*.

* P ≤ 0.05 and

** *P ≤ 0.01*.

*S1–S5 are as shown in Table 1. S|S-1 indicates the time interval from S-1 to S*.

Correlation analysis based on both unconditional and conditional SPAD values in RILs between different growth stages across water environments were given in Table 3. All correlations were positive and their correlation coefficients widely varied from 0.13 to 0.98^**^ across environments. Howbeit it appeared obvious that correlation coefficients decreased with the growth course, and even occurred weakly at the last stage. For most stages across water environments, unconditional correlations (*r*^2^ = 0.21~0.98^**^) were more significant and substantially higher than conditional ones (*r*^2^ = 0.13~0.51^**^). On the other hand as shown in Table 4, most of the correlations between unconditional and conditional data were poorly positive but rather low, with the exception of significant correlations occurring between the conditional period of S1|S0 and all unconditional stages, and between conditional periods and their corresponding unconditional stages. It was inferred that the greenness ontogeny of flag leaf was highly characteristic of dynamic and environmentally influenced scenarios.

**Table 3:** **Correlation coefficients of the greenness of flag leaf in RILs between different growth stages in different water environments**.

**Environment**	**Stage**	**S1/S1|S0**	**S2/S2|S1**	**S3/S3|S2**	**S4/S4|S3**	**S5/S5|S4**
E1	S1/S1|S0		0.96^**^/0.51^**^	0.84^**^/0.49^**^	0.68^**^/0.43^**^	0.35^*^/0.26
	S2/S2|S1	0.98^**^/0.49^**^		0.86^**^/0.45^**^	0.66^**^/0.23	0.28/0.21
	S3/S3|S2	0.90^**^/0.43^*^	0.92^**^/0.42^**^		0.67^**^/0.23	0.27/0.18
	S4/S4|S3	0.76^**^/0.37^*^	0.77^**^/0.38^*^	0.80^**^/0.31^*^		0.21/0.13
	S5/S5|S4	0.29/0.21	0.27/0.24	0.25/0.25	0.26/0.20	
E2	S1/S1|S0		0.92^**^/0.36^*^	0.71^**^/0.28	0.71^**^/0.44^**^	0.28/0.15
	S2/S2|S1	0.95^**^/0.40^*^		0.76^**^/0.24	0.64^**^/0.47^**^	0.25/0.24
	S3/S3|S2	0.83^**^/0.32^*^	0.86^**^/0.27		0.60^**^/0.39^*^	0.27/0.22
	S4/S4|S3	0.75^**^/0.33^*^	0.74^**^/0.23	0.76^**^/0.25		0.25/0.22
	S5/S5|S4	0.31/0.23	0.31/0.20	0.33/0.22	0.28/0.16	
E3	S1/S1|S0		0.92^**^/0.31	0.72^**^/0.21	0.71^**^/0.48^**^	0.27/0.18
	S2/S2|S1	0.94^**^/0.39^*^		0.74^**^/0.23	0.63^**^/0.41^*^	0.28/0.22
	S3/S3|S2	0.82^**^/0.35^*^	0.84^**^/0.27		0.61^**^/0.39^*^	0.25/0.21
	S4/S4|S3	0.72^**^/0.34^*^	0.72^**^/0.23	0.73^**^/0.20		0.22/0.15
	S5/S5|S4	0.28/0.20	0.26/0.19	0.26/0.21	0.21/0.17	

*The numbers at the left of the slash (“/”) are correlation coefficients for the unconditional phenotype of RILs, and the numbers at the right indicate the conditional phenotype of RILs*.

* P ≤ 0.05 and

** *P ≤ 0.01. Numbers in the upper right segment apply to the drought stress; those at the lower left are for the well-watered condition. E1–E3 and S1–S5 are as shown in Table 1, and S1|S0 to S5|S4 are as shown in Table 2*.

**Table 4:** **Correlation coefficients between unconditional and conditional greenness of flag leaf in RILs at five growth stages different water environments**.

**Environment**	**Stage**	**S1|S0**	**S2|S1**	**S3|S2**	**S4|S3**	**S5|S4**
E1	S1	1.00^**^/1.00^**^	0.31^*^/0.48^**^	0.33^*^/0.39^*^	0.37^*^/0.43^**^	0.25/0.30
	S2	0.96^**^/0.98^**^	0.47^**^/0.61^**^	0.36^*^/0.40^*^	0.38^*^/0.41^*^	0.24/0.31
	S3	0.84^**^/0.90^**^	0.36^**^/0.55^**^	0.51^**^/0.65^**^	0.38^*^/0.45^**^	0.22/0.30
	S4	0.68^**^/0.76^**^	0.34^*^/0.36^**^	0.47^**^/0.50^**^	0.53^**^/0.76^**^	0.24/0.26
	S5	0.36^*^/0.21	0.26/0.25	0.25/0.28	0.24/0.27	0.84^**^/0.89^**^
E2	S1	1.00^**^/1.00^**^	0.36^*^/0.40^*^	0.28/0.32	0.33^*^/0.43^**^	0.29/0.32
	S2	0.92^**^/0.95^**^	0.43^**^/0.50^**^	0.29/0.29	0.31/0.33	0.25/0.33
	S3	0.71^**^/0.83^**^	0.36^*^/0.37^*^	0.58^**^/0.58^**^	0.23/0.22	0.21/0.27
	S4	0.71^**^/0.75^**^	0.37^*^/0.43^**^	0.32/0.41^*^	0.60^**^/0.75^**^	0.26/0.30
	S5	0.28/0.23	0.27/0.32	0.21/0.24	0.21/0.26	0.82^**^/0.86^**^
E3	S1	1.00^**^/1.00^**^	0.31^*^/0.39^*^	0.21/0.35^*^	0.34^*^/0.48^**^	0.25/0.18
	S2	0.92^**^/0.94^**^	0.29^**^/0.51^**^	0.26/0.30	0.30/0.37^*^	0.23/0.30
	S3	0.72^**^/0.82^**^	0.33/0.45^**^	0.62^**^/0.63^**^	0.21/0.21	0.23/0.22
	S4	0.71^**^/0.72^**^	0.32/0.42^**^	0.37^*^/0.51^**^	0.63^**^/0.77^**^	0.28/0.28
	S5	0.27/0.24	0.21/0.29	0.22/0.37	0.21/0.39^*^	0.85^**^/0.88^**^

*The numbers at the left of the slash (“/”) are correlation coefficients estimated in the drought stress, and the numbers at the right indicate the well-watered condition*.

* P ≤ 0.05 and

** *P ≤ 0.01. E1–E3 and S1–S5 are as shown in Table 1, and S1|S0–S5|S4 are as shown in Table 2. Underline values mainly highlight in the higher and more significant correlation coefficients*.

### Unconditional QTL analysis for leaf greenness development

**Figure 1:**
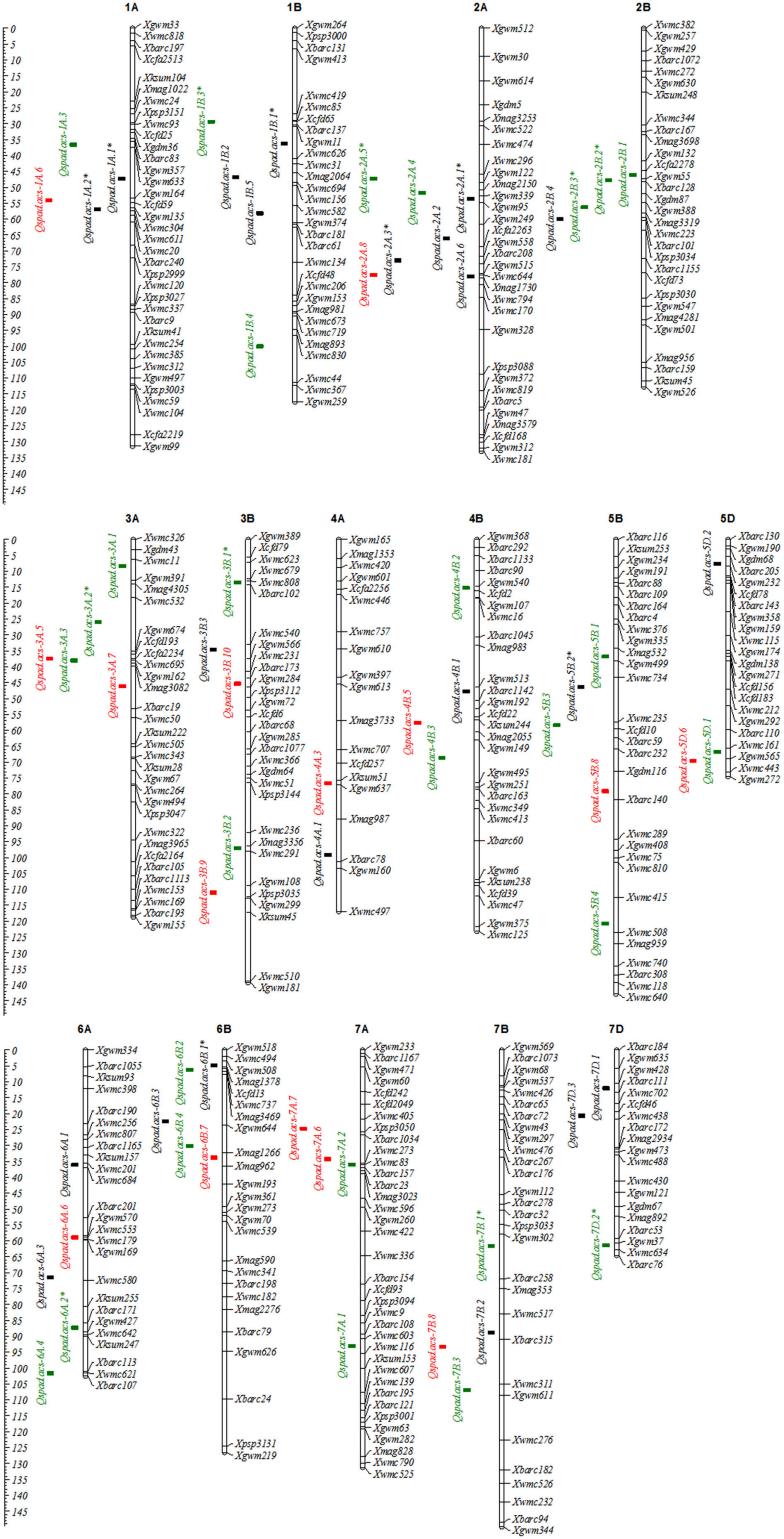
**Chromosome locations of additive QTLs for the greenness of flag leaf in wheat RILs**. Black squares are common QTLs for both unconditional and conditional chlorophyll content, and green and red squares are specific QTLs for unconditional and conditional chlorophyll content, respectively. QTLs symbolized with the asterisk (^*^) mean that QTLs can be detected more than two stages.

A total of 50 additive QTLs (A-QTLs) with significant *A* effects and/or *AE* effects were detected by unconditional SPAD data at different growth stages across water environments (Table 5). These loci were mapped on almost all chromosomes except 1D–4D, 5A, and 6D (Figure 1), individually explaining from 0.24 to 1.80% of the phenotypic variation. Half of them had positive *A* effects with 0.28^*^ to 0.94^***^ conferred by favorable alleles from Q9086, whereas the others with negative *A* effects of -0.26^*^ to -1.10^***^ were from Longjian 19. By contrast, a majority of putative A-QTLs (34 of 50) were identified at only one specific stage, but the remaining 16 loci were detectable at two to four stages, implying that no QTLs was continually active during the whole period of growth. For example, three of them, *Qspad.acs-1B.1, Qspad.acs-2A.3*, and *Qspad.acs-5B.2*, were related to four stages from S1 to S4. Two A-QTLs, *Qspad.acs-1A.2* and *Qspad.acs-6A.2*, were involved in three stages from S1 to S3 and from S2 to S4, respectively. The other 11 were just associated with two stages. Interestingly, these A-QTLs expressed at more than one stage were always conducted in the same sources of *A* effects, whereas effect values from each QTL progressively decreased with advanced stages. The number of A-QTLs per stage differed from 11 at S1 to 24 at the S3, and finally to 5 at S5. This suggested that expressions of A-QTLs governing leaf greenness were highly modulated by the developmental stage and were more activated in early periods. In addition, 27 A-QTLs showed significantly QEI effects, explaining from 0.87 to 5.42% of the phenotypic variations (Table 5), implying their genetic susceptibility to environments. Of these, about 90% of additive QEIs (A-QEIs) was highlighted in the period after S2, but few of them reacted at S1. During the period from S1 to S3, nine A-QTLs got involved in negative *AE* effects (−0.25^*^ to −1.03^***^) with the WW, but in positive effects (0.65^***^ to 1.12^***^) with the DS. This indicated that *AE* effects of these loci exposed during the early period could be more up-regulated by the DS. However, the cases for most of remainder loci expressed after S3 were opposite, with negative *AE* effects (−0.23^*^ to −0.87^***^) in the DS but positive effects (0.22^*^ to 0.85^***^) in the WW, suggestive of up-regulating them by the WW in the later duration.

**Table 5:** **Unconditional additive and interacting effects of QTL × water environment of identified QTLs for the greenness of flag leaf**.

***QTL***	**Flanking markers**	**Stage^a^**	***A*^b^**	***AE*^c^**	***H*^2^*(A)*^b^(%)**	***H*^2^*(AE)*^c^(%)**
*Qspad.acs-1A.1*	Xgwm633-Xgwm164	S1	0.81^***^		0.73	
		S2	0.72^***^		0.85	
*Qspad.acs-1A.2*	Xgwm135-Xwmc304	S1	0.76^***^		0.91	
		S2	0.65^***^		0.63	
		S3	0.48^***^	−0.66^***^(E2_DS_), −0.72^***^(E3_DS_), 0.78^***^(E2_WW_)	0.43	2.24
*Qspad.acs-1A.3*	Xgwm357-Xgwm633	S2	0.72^***^	−0.85^***^(E1_WW_)	0.87	2.54
*Qspad.acs-1B.1*	Xgwm11-Xwmc626	S1	−0.80^***^		0.63	
		S2	−1.10^***^	0.98^***^(E1_DS_), −0.92^***^(E3_WW_)	0.92	3.24
		S3	−0.76^***^		1.05	
		S4	−0.41^***^		1.31	
*Qspad.acs-1B.2*	Xmag2064-Xwmc694	S2	−0.76^***^	0.88^***^(E1_DS_), 0.83^***^(E3_DS_), −0.75^***^(E2_WW_)	0.82	1.64
*Qspad.acs-1B.3*	Xwmc85-Xcfd65	S2	−0.67^***^		0.68	
		S3	−0.45^***^		0.84	
*Qspad.acs-1B.4*	Xwmc830-Xwmc44	S4	0.59^***^	−0.31^**^(E1_DS_)	0.83	1.34
*Qspad.acs-1B.5*	Xwmc582-Xgwm374	S1	−0.59^***^		0.56	
*Qspad.acs-2A.1*	Xgwm339-Xgwm95	S1	0.77^***^	−0.93^***^(E2_WW_), −0.99^***^(E3_WW_)	1.05	1.71
		S3	0.46^***^	−0.56^***^ (E1_DS_)	0.69	0.87
*Qspad.acs-2A.2*	Xgwm249-Xcfa2263	S1	−0.57^***^		0.59	
*Qspad.acs-2A.3*	Xgwm558-Xbarc208	S1	0.94^***^	0.34^**^(E2_WW_)	1.06	1.14
		S2	0.84^***^	1.12^***^(E2_DS_), −1.03^***^(E2_WW_)	1.16	5.42
		S3	0.70^***^		1.80	
		S4	0.42^***^	−0.49^***^(E1_DS_), 0.40^***^(E2_WW_), 0.49^***^(E3_WW_)	0.92	1.91
*Qspad.acs-2A.4*	Xmag2150-Xgwm339	S2	0.75^***^		0.95	
*Qspad.acs-2A.5*	Xwmc296-Xgwm122	S4	0.49^***^	−0.56^***^(E1_DS_), 0.29^**^ (E2_WW_)	0.84	1.54
		S5	0.28^*^	−0.23^*^ (E3_DS_)	0.64	2.74
*Qspad.acs-2A.6*	Xwmc644-Xmag1730	S1	0.93^***^		1.29	
*Qspad.acs-2B.1*	Xcfa2278-Xgwm55	S2	0.64^***^		0.58	
*Qspad.acs-2B.2*	Xgwm55-Xbarc128	S2	0.78^***^		0.77	
		S3	0.61^***^	0.50^***^(E2_WW_)	0.72	2.09
*Qspad.acs-2B.3*	Xgwm388-Xmag3319	S3	0.65^***^		0.59	
		S4	0.49^***^		0.55	
*Qspad.acs-2B.4*	Xwmc223-Xbarc101	S2	0.83^***^		0.78	
*Qspad.acs-3A.1*	Xwmc11-Xgwm391	S2	−0.67^***^	0.78^***^(E2_DS_)	0.76	1.56
*Qspad.acs-3A.2*	Xwmc532-Xgwm674	S3	−0.55^***^	0.65^***^ (E3_DS_), −0.66^***^ (E2_WW_), −0.59^***^ (E3_WW_)	0.52	2.15
		S4	−0.41^***^		0.40	
*Qspad.acs-3A.3*	Xcfa2234-Xwmc695	S4	0.50^***^		0.71	
*Qspad.acs-3B.1*	Xwmc808-Xbarc102	S3	0.58^***^	−0.41^***^(E1_DS_), −0.45^***^(E2_DS_), 0.47^***^(E1_WW_)	0.42	1.66
		S4	0.44^***^		0.63	
*Qspad.acs-3B.2*	Xmag3356-Xwmc291	S3	0.61^***^		0.75	
*Qspad.acs-3B.3*	Xwmc540-Xgwm566	S1	−0.64^***^		0.75	
*Qspad.acs-4A.1*	Xmag987-Xbarc78	S5	−0.32^**^	−0.43^***^ (E2_DS_), −0.41^***^ (E3_DS_), 0.30^**^ (E1_WW_)	0.61	2.39
*Qspad.acs-4B.1*	Xgwm513-Xbarc1142	S4	−0.47^***^	−0.55^***^(E1_DS_), 0.25^*^ (E3_WW_)	0.37	1.58
*Qspad.acs-4B.2*	Xgwm540-Xcfd2	S4	0.62^***^		0.50	
*Qspad.acs-4B.3*	Xgwm149-Xgwm495	S3	0.48^***^		0.38	
*Qspad.acs-5B.1*	Xmag532-Xgwm499	S2	0.51^***^		0.44	
*Qspad.acs-5B.2*	Xwmc734-Xwmc235	S1	−0.88^***^	−0.25^*^(E3_WW_)	0.86	0.89
		S2	−0.76^***^		0.86	
		S3	−0.64^***^		1.11	
		S4	−0.49^**^	−0.52^***^ (E1_DS_), 0.30^**^(E3_WW_)	0.90	1.76
*Qspad.acs-5B.3*	Xwmc235-Xcfd10	S3	−0.67^***^		0.89	
*Qspad.acs-5B.4*	Xwmc415-Xwmc508	S3	0.45^***^		0.69	
*Qspad.acs-5D.1*	Xwmc161-Xgwm565	S4	−0.55^***^	−0.62^***^ (E2_DS_), −0.58^***^ (E3_DS_), 0.31^**^ (E1_WW_)	0.36	2.05
*Qspad.acs-5D.2*	Xbarc205-Xgwm232	S5	0.33^**^	−0.39^***^ (E2_DS_), 0.22^*^(E1_WW_)	0.47	1.91
*Qspad.acs-6A.1*	Xwmc201-Xwmc684	S2	0.35^**^	−0.39^***^(E2_WW_), 0.45^***^(E3_WW_)	0.49	1.56
*Qspad.acs-6A.2*	Xbarc171-Xgwm427	S2	−0.59^***^	0.28^*^(E2_WW_), 0.35^**^(E3_WW_)	0.28	1.26
		S3	−0.40^***^		0.36	
		S4	−0.39^***^		0.24	
*Qspad.acs-6A.3*	Xgwm169-Xwmc580	S3	−0.72^***^	−0.30^**^(E1_DS_), −0.33^**^(E3_DS_)	0.60	1.14
*Qspad.acs-6A.4*	Xbarc113-Xwmc621	S4	−0.69^***^	−0.72^***^(E2_DS_), −0.76^***^(E3_DS_), 0.39^***^(E3_WW_)	0.56	2.49
*Qspad.acs-6B.1*	Xgwm508-Xmag1378	S1	0.62^***^		0.70	
		S3	0.44^***^		0.38	
*Qspad.acs-6B.2*	Xcfd13-Xwmc737	S4	0.42^***^		0.28	
*Qspad.acs-6B.3*	Xmag3469-Xgwm644	S3	−0.51^***^		0.32	
*Qspad.acs-6B.4*	Xgwm644-Xmag1266	S3	−0.40^***^		0.25	
*Qspad.acs-7A.1*	Xwmc603-Xwmc116	S3	0.59^***^	0.73^***^(E2_DS_), 0.81^***^(E3_DS_), −0.72^***^(E2_WW_)	0.56	2.26
*Qspad.acs-7A.2*	Xwmc273-Xwmc83	S4	−0.45^***^		0.26	
*Qspad.acs-7B.1*	Xgwm302-Xbarc258	S3	−0.57^***^	−0.79^***^(E1_DS_), −0.72^***^(E3_DS_)	0.31	2.32
		S5	−0.39^***^		0.36	
*Qspad.acs-7B.2*	Xwmc517-Xbarc315	S3	−0.73^***^	−0.26^*^(E3_DS_), 0.29^**^(E3_WW_)	0.60	0.94
*Qspad.acs-7B.3*	Xwmc311-Xgwm611	S4	−0.62^***^		0.55	
*Qspad.acs-7D.1*	Xgwm428-Xbarc111	S3	−0.66^***^	−0.83^***^(E2_DS_), −0.80^***^(E3_DS_), 0. 85^***^(E1_WW_)	0.54	2.67
*Qspad.acs-7D.2*	Xgwm37-Xwmc634	S4	−0.43^***^	−0.53^***^(E1_DS_), −0.50^***^(E2_DS_), 0.51^***^(E2_WW_)	0.25	1.80
		S5	−0.26^*^	−0.30^***^(E3_DS_)	0.54	1.56
*Qspad.acs-7D.3*	Xcfd46-Xwmc438	S3	−0.73^***^	−0.87^***^(E1_DS_), −0.80^***^(E2_DS_)	0.68	2.52

a *1–S5 are as shown in Table 1*.

b A, the additive effect. A positive value indicates the genetic effect from Q9086 allele, and a negative value represents the genetic effect from Longjian 19 allele;

* *P ≤ 0.01*,

** P ≤ 0.005, and

*** *P ≤ 0.001; H^2^(A) (%) indicates the proportion of phenotypic variance explained by additive QTL*.

c *AE, the additive QTL × environment interaction effects in drought stress (DS) and the well-watered (WW) conditions in E1–E3 shown in Table 1. H^2^(AE)(%) indicates the phenotypic variance explained by additive QTL × environment interaction*.

All 38 pairs of epistatic QTLs (AA-QTLs) for the greenness of flag leaf were available with significant *AA* effects, accounting for phenotypic variations of 1.08–3.29% in response to different stages and environments (Table S1). These loci involved in epistasis were distributed on all chromosomes apart from 1B and 5A. Among them, 13 pairs behaved with positive *AA* effects (0.82^***^ to 1.32^***^), indicating that parent-type effects were higher than recombinant-type effects. And, 23 pairs showed negative effects (−0.42^***^ to −1.51^***^) where recombinant-type effects were higher than parent-type effects. The remaining two pairs altered in effect directions responsive to the specific stage, where one pair had positive effects (0.78^***^) at S4 but negative effects (−0.45^***^) at S5, and another pair showed positive effects (1.34^***^) at S1 but negative effects (−0.93^***^ and −0.75^***^) at S2 and S3, respectively. Similar to the expression patterns of A−QTLs, putative AA-QTLs were also expressed dynamically and alternatively. For example, the amounts of significant AA-QTLs for each stage subsequently decreased, ranging from 24 at the S1 to 2 at the S5. Moreover, 20 pairs were detected at only one specific stage, while the other pairs were identified more at two to three early consecutive stages (S1 to S2 or S3) except three pairs available after S3. Thus, epistatic reactions to the ontogeny of leaf greenness were short lived without more genetic effects appearing in the later duration, of which *AA* effects progressively decreased. Apart from *AA* effects, significant *AAE* effects were also involved in 24 AA-QTLs, explaining phenotypic variations of 0.82–2.54%. Over 76% of epistatic QEIs (E-QEIs) reacted in the period after S2. Of these, just two pairs involved positive *AAE* effects (0.66^***^ and 0.52^***^, respectively) with the DS. The other 22 pairs entirely specified their *QEI* with negative effects (−0.33^**^ to −0.95^***^) in the DS, while positive effects (0.31^**^ to 0.94^***^) in the WW. This indicated that *AAE* effects were highly up-regulated by the WW.

**Figure 2:**
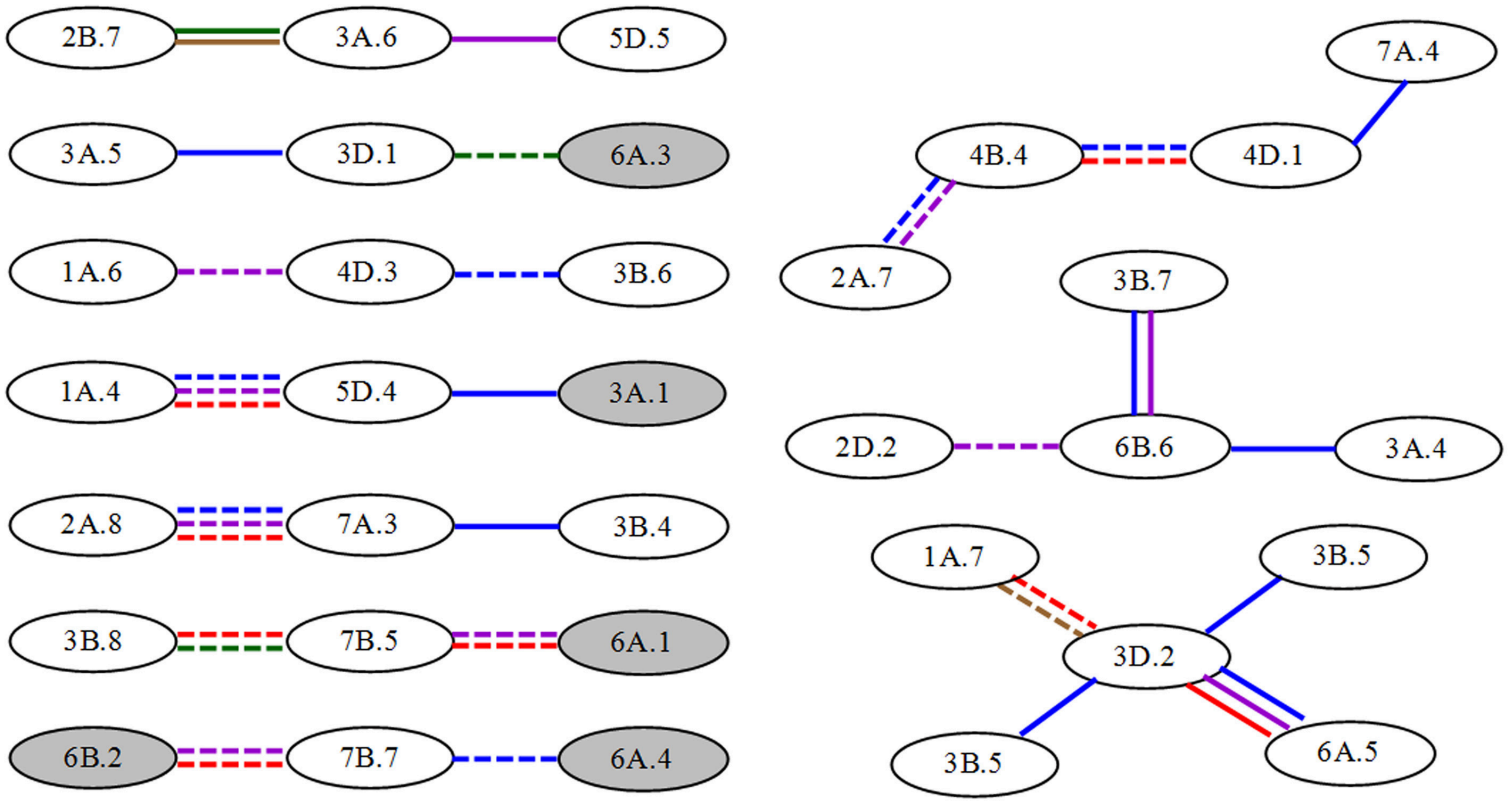
**Epistatic QTL network for unconditional greenness of flag leaf at different stages in wheat RILs**. Gray and white ellipses indicate A-QTLs and non-individual QTLs, respectively. Solid and dashed lines represent QTL interactions with positive and to negative *AA* effects, respectively. The different colors such as blue, purple, red, green and brown mean the stages from S1 to S5, respectively.

Concerning the source of epistatic loci, only 10 significant A-QTLs participated in epistatic interactions and therefore exhibited their pleiotropic functions. Howbeit most of epistatic interactions (nearly 90%) were derived from non-individual QTLs, which were involved in epistatic QTLs without any significant *A* effects. These loci even constituted QTL-interacting networks at different interaction levels (Figure 2) to realize different *AA* effects. For instance, five A-QTLs interacting with 16 non-individual loci were composed of seven smaller networks by three-locus interactions, respectively. The remaining 13 non-individual QTLs made up three relatively bigger networks from four or five-locus interactions, respectively. Almost 60% of interactions exhibited negative *AA* effects with unequal magnitudes at one to three stages. This further suggested that the genetic control of the greenness development of flag leaf was complex and, to a certain extent, reacted as part of QTL networks.

### Conditional QTL analysis for leaf greenness development

Based on conditional mapping, a total of 37 A-QTLs were identified with significant *A* effects and/or *QEI* effects for the greenness ontogeny of flag leaf across water environments (Table 6). These loci were nearly distributed on the same chromosomes as unconditional ones (Figure 1), individually explaining from 0.36 to 1.29% of the phenotypic variation. Of these, nearly half of favorable alleles derived from Q9086 with significantly positive *A* effects (0.30^**^ to 0.94^***^), whereas another half came from Longjian 19 with significantly negative effects (−0.31^**^ to −0.88^***^). The result was exactly consistent with the finding from unconditional mapping, further confirming that favorable alleles were averagely dispersed within the two parents. All of conditional A-QTLs were available just in one specific period. The QTL number detected in each period gradually reduced from 11 at S1 to 2 at S5. This indicated that genes governing leaf greenness were expressed selectively but more in early development period. In addition, 18 loci were noticeably associated with A-QEIs, individually accounting for 0.82–3.80% of the phenotypic variation. Most of them (nearly 85%) happened in the duration from S2|S1 to S5|S4, suggesting that the real gene expression in the period of S1|S0 was less influenced by water environments, whereas it was extremely done thereafter. In this regards, all of A-QTLs interacting with the DS showed significantly negative *AE* (−0.32^**^ to −0.79^***^) in different periods across environments. By contrast, most of them reacting to the WW across environments exhibited significantly positive *AE* (0.25^*^ to 0.80^***^), with exception of two QTLs, *Qspad.acs-2A.1* and *Qspad.acs-5B.2*, behaving significantly negative *AE* (−0.25^*^ to −0.99^***^) in S1|S0. This indicated that the net expression of one locus in the specific period was sensitive to water supply with alternative effect directions, and generally highlighted in up-regulation by the WW.

**Table 6:** **Conditional additive and interacting effects of QTL × environment of identified QTLs for the greenness of flag leaf**.

***QTL***	**Flanking markers**	**Stage^a^**	***A*^b^**	***AE*^c^**	***H*^2^*(A)*^b^(%)**	***H*^2^*(AE)*^c^(%)**
*Qspad.acs-1A.1*	Xgwm633-Xgwm164	S1|S0	0.81^***^		0.73	
*Qspad.acs-1A.2*	Xgwm135-Xwmc304	S1|S0	0.76^***^		0.91	
*Qspad.acs-1A.6*	Xgwm164-Xcfd59	S2|S1	0.64^***^		0.66	
*Qspad.acs-1B.1*	Xgwm11-Xwmc626	S1|S0	−0.80^***^		0.63	
*Qspad.acs-1B.2*	Xmag2064-Xwmc694	S3|S2	0.59^***^	0.39^***^(E2_WW_), 0.36^***^(E3_WW_)	0.60	1.08
*Qspad.acs-1B.5*	Xwmc582-Xgwm374	S1|S0	−0.59^***^		0.56	
*Qspad.acs-2A.1*	Xgwm339-Xgwm95	S1|S0	0.77^***^	−0.93^***^(E2_WW_), −0.99^***^(E3_WW_)	1.05	1.71
*Qspad.acs-2A.2*	Xgwm249-Xcfa2263	S1|S0	−0.57^***^		0.59	
*Qspad.acs-2A.3*	Xgwm558-Xbarc208	S1|S0	0.94^***^	0.34^**^(E2_WW_)	1.06	1.14
*Qspad.acs-2A.6*	Xwmc644-Xmag1730	S1|S0	0.93^***^		1.29	
*Qspad.acs-2A.8*	Xgwm515-Xwmc644	S4|S3	−0.47^***^	0.25^*^(E1_WW_)	0.49	0.82
*Qspad.acs-2B.4*	Xwmc223-Xbarc101	S3|S2	−0.58^***^		0.50	
*Qspad.acs-3A.5*	Xcfd193-Xcfa2234	S2|S1	0.61^***^		0.84	
*Qspad.acs-3A.7*	Xmag3082-Xbarc19	S3|S2	−0.72^***^		0.99	
*Qspad.acs-3B.3*	Xwmc540-Xgwm566	S1|S0	−0.64^***^		0.75	
*Qspad.acs-3B.9*	Xgwm108-Xpsp3035	S2|S1	0.53^***^	0.52^***^(E2_WW_), 0.54^***^(E3_WW_)	0.76	2.05
*Qspad.acs-3B.10*	Xbarc173-Xgwm284	S2|S1	0.69^***^	−0.43^***^(E2_DS_)	0.63	1.22
*Qspad.acs-4A.1*	Xmag987-Xbarc78	S2|S1	−0.36^**^	−0.76^***^(E1_DS_), −0.78^***^(E3_DS_)	0.40	3.80
*Qspad.acs-4A.3*	Xksum51-Xgwm637	S2|S1	−0.54^***^		0.73	
*Qspad.acs-4B.1*	Xgwm513-Xbarc1142	S4|S3	−0.39^**^	−0.76^***^(E2_DS_), −0.70^***^(E3_DS_), 0.75^***^(E3_WW_)	0.36	3.25
*Qspad.acs-4B.5*	Xksum244-Xmag2055	S3|S2	−0.31^**^	−0.77^***^(E2_DS_), −0.78^***^(E3_DS_), 0.72^***^(E2_WW_)	0.41	3.19
*Qspad.acs-5B.2*	Xwmc734-Xwmc235	S1|S0	−0.88^***^	−0.25^**^(E3_WW_)	0.86	0.89
*Qspad.acs-5B.8*	Xgdm116-Xbarc140	S2|S1	−0.62^***^		0.74	
*Qspad.acs-5D.2*	Xbarc205-Xgwm232	S3|S2	0.33^**^	−0.41^***^(E1_DS_), 0.32^**^ (E3_WW_)	0.39	2.72
*Qspad.acs-5D.6*	Xgwm565-Xwmc443	S4|S3	0.41^***^	−0.32^**^(E2_DS_), 0.26^*^ (E3_WW_)	0.75	3.52
*Qspad.acs-6A.1*	Xwmc201-Xwmc684	S2|S1	0.73^***^		0.86	
*Qspad.acs-6A.3*	Xgwm169-Xwmc580	S4|S3	−0.48^***^	−0.41^***^(E2_DS_), 0.47^***^(E3_WW_)	0.49	2.53
*Qspad.acs-6A.6*	Xgwm570-Xwmc553	S3|S2	−0.55^***^		0.66	
*Qspad.acs-6B.1*	Xgwm508-Xmag1378	S1|S0	0.62^***^		0.70	
*Qspad.acs-6B.3*	Xmag3469-Xgwm644	S5|S4	−0.46^***^		0.40	
*Qspad.acs-6B.7*	Xmag1266-Xmag982	S3|S2	−0.43^***^		0.38	
*Qspad.acs-7A.6*	Xbarc1034-Xwmc273	S4|S3	0.39^***^	−0.41^***^(E2_DS_), 0.21^*^(E2_WW_)	0.74	2.63
*Qspad.acs-7A.7*	Xwmc405-Xpsp3050	S3|S2	0.48^**^	−0.46^***^(E1_DS_), 0.34^**^(E1_WW_)	0.36	2.80
*Qspad.acs-7B.2*	Xwmc517-Xbarc315	S5|S4	0.30^**^	−0.33^**^(E2_DS_)	0.57	2.31
*Qspad.acs-7B.8*	Xbarc315-Xwmc311	S2|S1	−0.63^***^		0.81	
*Qspad.acs-7D.1*	Xgwm428-Xbarc111	S3|S2	0.49^***^	−0.49^***^(E1_DS_), −0.47^***^(E2_DS_), 0.47^***^(E3_WW_)	0.59	1.68
*Qspad.acs-7D.3*	Xcfd46-Xwmc438	S2|S1	−0.34^**^	−0.75^***^(E1_DS_), −0.79^***^(E3_DS_), 0.80^***^(E1_WW_)	0.51	3.01

a *S1|S0–S5|S4 are as shown in Table 2*.

b A, the additive effect. A positive value indicates the genetic effect from Q9086 allele, and a negative value represents the genetic effect from Longjian 19 allele;

* *P ≤ 0.01*,

** P ≤ 0.005, and

*** *P ≤ 0.001; H^2^(A) (%) indicates the proportion of phenotypic variance explained by additive QTL*.

c *AE, the additive QTL × environment interaction effects in drought stress (DS) and the well-watered (WW) conditions in E1–E3 shown in Table 1. H^2^(AE)(%) indicates the phenotypic variance explained by additive QTL × environment interaction*.

Total of 29 epistatic pairs were mapped on the nearly same chromosomes as unconditional loci other than chromosome 1D, accounting for from 0.66 to 3.29% of the phenotypic variation (Table S2). Of these, 10 pairs appeared significantly positive *AA* effects (0.72^***^ to 1.32^***^), whereas the other 19 pairs behaved significantly negative effects (−0.58^***^ to −1.51^***^). Apart from one pair detectable in two continual periods (S2|S1 and S3|S2), the other 28 pairs were identified in a specific period, especially in S1|S0—i.e., 25 pairs available. This further confirmed that epistatic effects were short lived but highly predominant in early period. In addition, 14 pairs got involved in E-QEIs, explaining from 0.86 to 4.08% of the phenotypic variation. Nearly 43% of them occurred in S1|S0, the remainders were involved in other periods. Although expressional patterns of AA-QTLs differed from one to another period in response to water environments, all of them exhibited negative effects (−0.54^***^ to −0.95^***^) in the DS, but positive effects (0.52^***^ to 0.98^***^) in the WW. This indicated that the expressions of conditional AA-QTLs were also enhanced by the WW, in accordance with results of the unconditional analysis.

**Figure 3:**
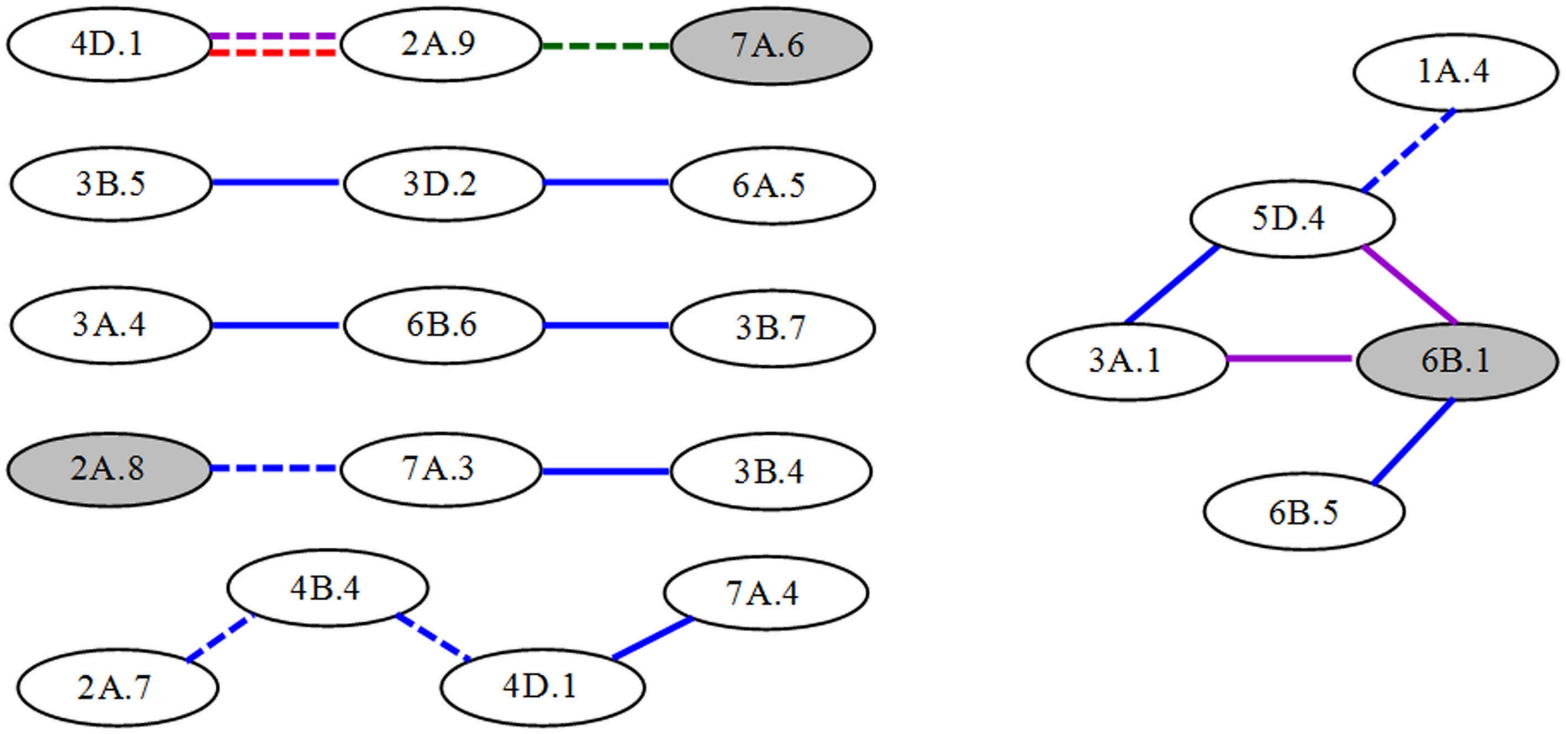
**Epistatic QTL network for conditional greenness of flag leaf at different stages in wheat RILs**. Gray and white ellipses indicate A-QTLs and non-individual QTLs, respectively. Solid and dashed lines represent QTL interactions with positive and to negative *AA* effects, respectively. The different colors such as blue, purple, red and green mean the stages from S1|S0 to S4|S3, respectively.

For the compositions of conditional epistatic loci, most of epistatic interactions were performed by non-individual QTLs, besides seven significant A-QTLs involving epitasis. Thus, QTL networks were involved at different interaction levels (Figure 3), but simpler than those of unconditional AA-QTLs. For example, two significant A-QTLs and 10 non-individual QTLs were participated in four three-locus-interaction networks. One A-QTL and other eight non-individual QTLs were involved in two bigger networks from four or five-locus interactions, respectively. In these QTL networks, most of epistatic interactions (nearly 65%) had positive *AA* effects and more occurred in S1|S0, indicative of predominance in parent-type effects and in time-independent expressions in the first period.

### Comparative analysis between conditional and unconditional QTLs

Following the above mapping results, 22 A-QTLs (Tables 5, 6) and 25 pairs of AA-QTLs (Tables S1, S2) were common between unconditional and conditional mapping strategies across five measuring stages. With regard to these common loci, 11 A-QTLs and 24 AA-QTLs were detected at the first stage, where both genetic effects and contribution rates for the phenotypic variation were exactly equal to conditional ones. However, the remainder loci expressed at one or more stages, responding to specific stages and water environments, remarkably differed in *A* and *AE* effects, as well as in their contribution rates between two sets of mapping strategies. This suggested that common loci also behaved alternatively for inheritance of leaf greenness, with cumulative or net genetic effects highly modulated by developmental courses and water environments. Opposite to these common loci, 28 A-QTLs and 13 pairs of AA-QTLs were specifically detected by the unconditional mapping, whereas, in this way, 15 A-QTLs and 4 pairs of AA-QTLs were identified only by the conditional mapping. Therefore, by combining these two sets of mapping strategies based on time-dependent evaluation, more novel loci might be available and further exposed dynamic expression of polygenes governing the ontogeny of leaf greenness. Furthermore, an interesting feature was that both common and specifically-expressed A-QTLs detected by two sets of mapping strategies were nearly distributed in cluster occurred in specific neighboring marker intervals in several chromosomes (Figure 1). For example, two to seven loci shared neighboring intervals with flanking markers from Xgwm357 to Xwmc304 on chromosome 1A, from Xwmc85 to Xgwm374 on chromosome 1B, from Xwmc 296 to Xmag1730 on chromosome 2A, and so on, respectively. This indicated that specific marker intervals might carry a wealth of genetic information for the ontogeny of leaf greenness.

**Figure 4:**
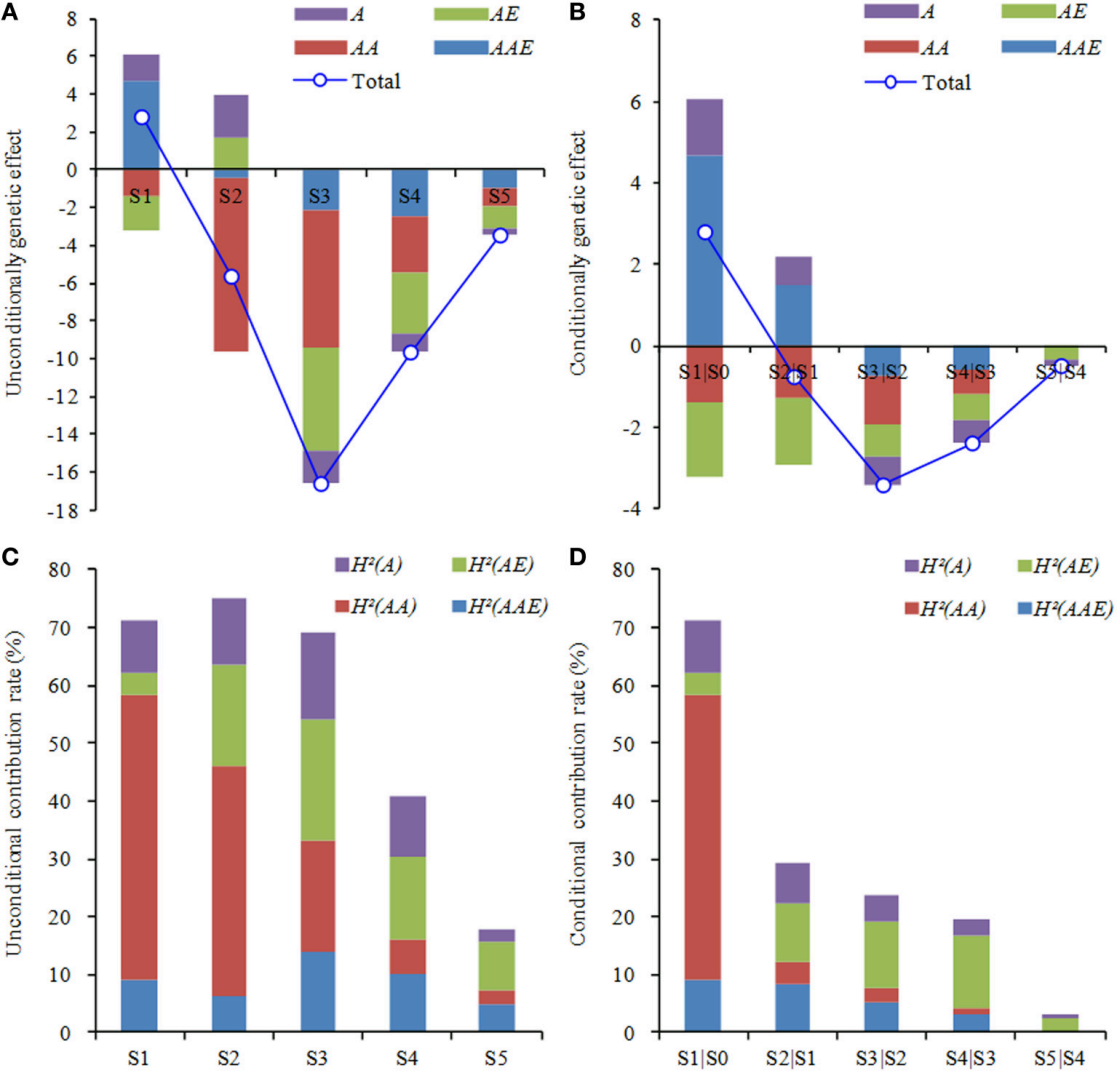
**Components of genetic effects (A,B) and contribution (C,D) to development of the greenness of flag leaf in wheat RILs**. *A*, the additive effect; *AE*, the additive QTL × environment interaction effects; *AA*, the epistatic effect; and *AAE*, the interaction effect of epistatic QTL × environment. *H*^2^(*A*), *H*^2^(*AE*), *H*^2^(*AA*), and *H*^2^(*AAE*) indicate the phenotypic variance explained by corresponding genetic effect. S1–S5 are as shown in Table 1, and S1|S0 to S5|S4 are as shown in Table 2.

With regard to the general effects of genetic components from two sets of mapping analysis, the dynamics of QTL expressions was highly visible in the measuring duration of ontogeny (Figures 4A,B). The general effects almost appeared negative, but their absolute values greatly altered in different periods, as the trend in noticeable increase before the third phase and thereafter decrease to the minimum in the final period. Except the equal effect values emerging in the first period, the other effect values of unconditional QTLs were considerably higher than those of conditional ones. It could be perceived that the genetic regulation for the development of leaf greenness by cumulative effects was stronger than that by net effects. On the other hand, both unconditional and conditional *AAE* effects were greatly higher than other genetic components in the first period. During the second period, superior genetic components were associated with *AA* effects in unconditional mapping, but *AE* and *AAE* in conditional mapping. Thereafter, superior genetic components almost tended to the similarity between two sets of mapping strategies, highlighting in *AA* and/or *AE* effects. This suggested that, in each developmental period, QTLs governing the ontogeny of leaf greenness were also expressed dynamically, ascribed to the specific effect strength of genetic components.

On the other hand, the general contribution rates explaining the phenotypic variation were also further illustrated the dynamic characteristics of QTL expressions for ontogeny of leaf greenness (Figures 4C,D). The trend appeared decline in whole measuring duration, whereas drift magnitudes were less in unconditional QTLs than conditional ones. During the early period (S1–S3), unconditional QTLs showed higher general contribution rates (69.06–75.24%), and then decreased to the bottom (17.82%) at last stage. However, the maximum (71.23%) of conditional loci happened in S1|S0, and then sharply declined to the minimum (3.28%) in S5|S4. This indicated that cumulative genetic effects could be maintained longer and stronger activation, whereas net genetic effects were weaker and short lived. By contrast to the contribution rates of genetic components, the predominant performances were mainly attributed to *AA* effects at S1–S2 or *AE* effects at S3–S5 by the unconditional mapping, and *AA* effects in S1|S0 or *AE* effects in S2|S1 to S5|S4 by the conditional mapping. Owing to the above analysis, both genetic effects and their contribution rates altered dynamically with a similar variation trend in progressive reduction during the whole measuring period, but mainly expressed in the early duration. Furthermore, the performances of *AA* or *AE* effects were highly predominant to determine the developmental genetics of leaf greenness.

## Discussion

According to the theory of developmental genetics, functional genes will be expressed dynamically in response to different growth stages. Furthermore, the expression pattern essentially occurs through the actions and interactions of polygenes during the ontogeny and is also flexibly modified by environments (Atchley and Zhu, 1997; Wu et al., 1999). Thus, the combination of unconditional with conditional mapping is verified to be an efficient approach to dissect dynamic expressions of developmental QTLs and reveal the inheritance of quantitative traits (Wu et al., 2010; Li S. et al., 2012). The strategy has been successfully applied to developmental QTLs analysis for agronomic and physiological traits in many crops (Liu et al., 2010; Wu et al., 2010; Han et al., 2011; Li S. et al., 2012). So far, although a multitude of previous studies have identified a wealth of QTLs for leaf staygreen and its associated traits by the method of unconditional mapping (Verma et al., 2004; Kumar et al., 2010; Li H. et al., 2012; Naruoka et al., 2012; Barakat et al., 2013; Czyczyło-Mysza et al., 2013), it seems rather obscure on genetic information of the ontogeny of leaf staygreen in wheat, because of the complexity. In this study, by employing unconditional and conditional mapping, a total of 65 A-QTLs and 42 pairs of AA-QTLs for the ontogeny leaf staygreen after flowering were detected on almost all 21 chromosomes except 5A across water environments, individually explaining from 0.24 to 3.29% of the phenotypic variation, indicative of typical quantitative traits controlled by minor-effect polygenes. Regardless of cumulative or net genetic effects, all loci were highly characteristic of time-dependent expressions. In this context, most of them were associated with specific development stage, while no locus was continually detectable over measuring time, except few loci active in two or more stages. Obviously, more QTLs were detected in earlier development stages and showed higher performances of genetic effects (Figure 4). The selective expressions of QTLs might be favorable to the complicated genetic regulations responsible for the ontogeny (Wu et al., 1999, 2010; Li S. et al., 2012). Likewise, the result could interpret why the phenotypic values of leaf greenness varied in subsequently decreased trend during the measuring period (Table 1). It was confirmed that the maintenance of leaf greenness might be greatly dependent upon early-expressed QTLs. And in another aspect, some major QTLs could be easily neglected in case only evaluating them by the phenotypic data at a specific stage, especially in the later period. Similar findings were also observed in wheat plant height (Wu et al., 2010), soybean pod number (Sun et al., 2006), and rice tiller trait (Yang et al., 2006).

By contrast, a total of 22 A-QTLs and 25 pairs of AA-QTLs were common between two sets of mapping methods. Besides, specifically- and reproducibly-expressed QTLs were more detected by the unconditional analysis (Table 5, Table S1). Each reproducibly-expressed A-QTL or AA-QTL showed significant genetic effects with same effect directions. For instance, one A-QTL, *Qspad.acs-1B.1*, showed negative *A* effects (−0.41^***^ to −1.10^***^) across four stages (S1–S4). One pair, *Qspad.acs-3D.2* × *Qspad.acs-6A.5*, exhibited positive *AA* effects (1.00^***^ to 1.32^***^) across three stages (S1–S3). This could be explained by the fact that unconditional QTLs were attributed to the cumulative expression from the initial time to stage *t* (Zhu, 1995). On the other hand, conditional QTLs *per se* could interpret the real gene expression in the specific period from stage *t-*1 to *t* (Zhu, 1995; Atchley and Zhu, 1997). Here almost all conditional QTLs were expressed only in a specific period (Table 6, Table S2). Other than QTLs detected at the first stage, conditional loci were significantly distinct from unconditional ones—e.g., some loci were observed with unconditional effects but without any conditional effect, and vice versa. Even though there existed some common QTLs between two mapping strategies, their expression profiles were variable in response to the specific measuring stage. For example, *Qspad.acs-1B.2* showed the unconditional *A* effect with -0.76^***^ at S2, but the conditional effect with 0.59^***^ in S3|S2. This indicated that parental contribution of favorable alleles at the same map position was also variable along with the development of leaf greenness. Therefore, the above evidence clearly suggests that QTL expressions for the ontogeny of leaf greenness are time-dependent. By combining unconditional QTL mapping with conditional QTL one of time-dependent measures, it is quite possible to reveal the dynamic gene expressions for the development of leaf staygreen.

The ability of a genotype to adapt its phenotype to different environments is referred as phenotypic plasticity (Ungerer et al., 2003). The phenotypic plasticity of quantitative traits arises in nature from QEIs at molecular levels (Campbell et al., 2003). Several examples of QEIs for developmental traits showed that the expression of particular chromosome regions differs across environments (Wu et al., 2010; Li S. et al., 2012). In this study, 53.0% of A-QTLs and 62.8% of AA-QTLs for the ontogeny of leaf greenness were significantly interacted with water environments, explaining from 0.82 to 5.42% and from 0.82 to 4.08% of the phenotypic variation, respectively. This indicated that the expressions of QTLs governing the ontogeny of leaf greenness were more susceptible to water environments, and to a certain extent, environmentally dependent. As for the attributes of QTL expressions influenced by water availability, there were significant differences among different developmental stages and between two mapping strategies. Firstly, although all of QEIs reacted to at least one water environment, the stages responsive to QEIs were widely various. Generally, most of them were highlighted in the periods after the first stage. For example, about 90% of unconditional A-QEIs and 85% of conditional A-QEIs flexibly occurred in the mid-anaphase, indicating that water environments highly affected the expressions of developmental QTLs in the later period of growth. Secondly, more QEIs occurred in unconditional QTLs than in conditional ones. For example, 27 A-QEIs and 24 E-QEIs were detected by the unconditional QTL mapping, where only 18 and 14 QEIs were unraveled by the conditional QTL mapping. Especially in the unconditional QTL mapping, four A-QEIs and seven E-QEIs had continually-expressed *AE* or *AAE* effects at two or three stages. However, the similar result was observed with only one E-QEI in the conditional QTL mapping. Of course, QEI effects were thus greatly distinct, except the QEIs involved in the first stage. This indicated that cumulative genetic effects were more prone to interact with water environments than net genetic effects. Thirdly, the behaviors of QEIs differed from responses to specific water environments. For example, nine unconditional A-QTLs expressed during the period from S1 to S3 got involved in negative *AE* effects with WW environments, but positive effects with DS environments. Thereafter, the case was opposite. This indicated that *AE* effects of these loci exposed during the early period could be more up-regulated by the DS. However, the other QEIs showed negative interaction effects with the DS, but positive effects with the WW. This clearly suggested that putative QTL expressions for the ontogeny of leaf greenness were environmentally-dependent and significantly up-regulated by WW environments. These results could provide detailed information on the variable performance of quantitative loci controlling the development of leaf greenness under different water environments.

Regarding the genetic components of leaf staygreen in wheat, several previous studies were elucidated that additive effects are predominant (Silva et al., 2000; Verma et al., 2004; Joshi et al., 2007; Kumar et al., 2010). In some cases, epistatic effects (Zhang et al., 2009a,b, 2010; Kumar et al., 2012) and QEI effects (Yang et al., 2007; Peleg et al., 2009) were also considered to be important. However, current genetic gains for leaf staygreen trait are made only by the traditional mapping analysis depending on phenotypic data at one time point. Indeed, it is inadequate to deeply dissect genetic components and their dynamical behaviors for the ontogeny of leaf staygreen. In the present study, the genetic behaviors for respective components were obviously dynamical and time-dependent during the whole measuring stages (Figure 4). Of these, general effects were almost negative, but had a large alternation in the third duration, which might essentially illustrate why phenotypic values of leaf greenness always decreased in the duration of growth, along with significant reduction around the stage of S3 across environments (Table 1). In view of the respective effects of genetic components, *AA* and QEIs effects were superior to other genetic components by two sets of mapping analysis, while effect directions and magnitudes varied in response to specific periods. The similar result was also observed in the developmental behavior of rice tiller number (Liu et al., 2010). On the other hand, the general contribution rates showed progressive reduction over the measuring time (Figure 4), consistent with the findings from the developmental genetic attributes of plant height (Wang et al., 2010; Wu et al., 2010) and grain weight (Li S. et al., 2012) in wheat. Thus, genetic effects active in early stages might play a critical role in modulating the phenotypic variation of development traits, due to higher contribution rates. Similar to the variations of genetic effects, the respective contribution rates of genetic components were highly flexible, whereas their magnitudes were incompletely equal to those of genetic effects. It was considered that some specific effects might be counteracted or pyramided each other during the development of quantitative traits (Wu et al., 2010). Nevertheless, the contribution rates of *AA* and *AE* effects were more predominant than other effects. The finding is confirmed that the action a specific gene to one quantitative phenotype is the collective property of a network of polygenes and/or its tight interactions with environments, rather than the behavior of a single gene (Wade, 2002; Malmberg et al., 2005).

In this study, although putative A-QTLs were widely dispersed on almost all chromosomes except 1D–4D, 5A, and 6D, they were nearly concentrated in specific neighboring marker intervals in several chromosomes. Moreover, these important marker intervals harbored many reproducibly-expressed QTLs in two or more periods (Figure 1). Using a wheat microsatellite consensus map (Somers et al., 2004) as a reference map, some QTLs controlling leaf greenness in the present work have been previously mapped on similar chromosomal regions. For example, four A-QTLs were detected in the marker interval from Xgwm357 to Xwmc304 on chromosome 1A, which overlapped with the location of a staygreen QTL (*Qsg.bhu-1A*) reported by Kumar et al. (2010). The reproducibly-expressed QTL, *Qspad.acs-1B.1*, shared the similar interval (Xgwm11–Xwmc626) of putative loci controlling leaf chlorophyll content (Czyczyło-Mysza et al., 2013). Another reproducibly-expressed QTL, *Qspad.acs-2A.1*, was possibly equal to *Qchl a+b.igdb-2A* (Li H. et al., 2012), as both were very close to the marker Xgwm339. Likewise, the other loci, such as *Qspad.acs-2B.1, Qspad.acs-3B.2, Qspad.acs-4A.3, Qspad.acs-5B.1, Qspad.acs-5D.2, Qspad.acs-7B.3, Qspad.acs-7D.1*, and *Qspad.acs-7D.2*, were identical or adjacent to the corresponding loci governing leaf chlorophyll content or its component content observed in different wheat populations (Zhang et al., 2009a; Kumar et al., 2010; Li H. et al., 2012; Czyczyło-Mysza et al., 2013). These common QTLs and their tightly-linked molecular markers would be of great importance for MAS. By contrast to current results reported by Czyczyło-Mysza et al. (2013), some typical marker intervals harboring QTLs clusters especially on chromosomes of 2B, 3A, 4B, 5B, 6B, and 7A (Figure 1) were also co-located considerable loci related to chlorophyll fluorescence parameters, carotenoid content, and even agronomic traits. For example, in the marker interval from Xwmc494 to Xgwm193 on chromosome 6B, five A-QTLs were mapped in this study, in which loci for related traits, such as amount of excitation energy trapped in PSII reaction centers (ET_0_/CS_m_), overall performance index of PSII photochemistry (PI), the maximum photochemical efficiency (Fv/Fm), light energy absorption (ABS/CS_m_), and grain yield for the main stem (GWE), were also located. Another marker interval from Xwmc11 to Xbarc19 harboring five loci for leaf greenness was possibly equivalent to the QTL-rich interval from Xwmc11 to Xcfa2262, where Czyczyło-Mysza et al. (2013) identified several QTLs controlling leaf carotenoid content, ET_0_/CS_m_, number of active reaction centers (RC/CS_m_), and GWE. This indicated that the hot-spot regions of QTLs could carry a wealth of genetic information on leaf greenness and its associated traits of wheat. Therefore, further studies on the possibility of a tight linkage or genetic pleiotropism on the QTL-rich regions will be very important, so as to elucidate the genetic nature of leaf greenness, and to use them in wheat improvement program.

## Conclusion

Flag leaf greenness of wheat in reproductive phase was controlled by minor-effect polygenes, which were expressed selectively as a time- and environmentally-dependent pattern during ontogeny. No single QTL was continually active in measuring period. But more loci were identified in early development periods, showing the higher performance of genetic effects. QEIs mainly happened in the mid-anaphase of development, where drought stress was more conducted with negative regulation on the QTL expressions. By contrast, *AA* and *AE* effects could be predominant in regulating phenotypic variations during the ontogeny of leaf greenness. In this regards, cumulative genetic effects could be maintained longer and stronger activation, whereas net genetic effects were weaker and short lived. Several QTL cluster regions were suggestive of tight linkage or expression pleiotropy in the inheritance of these traits. Some reproducibly-expressed QTLs or common loci consistent with previously detected would be useful to the genetic improvement of staygreen types in wheat through MAS, especially in water-deficit environments.

## Author contributions

DY designed the whole experiments and wrote the manuscript. ML and YL performed statistic analysis. LC, JC, and HC accomplished the phenotypic observation and measurement. SC performed the management of field experiments. All authors have read the manuscript.

### Conflict of interest statement

The authors declare that the research was conducted in the absence of any commercial or financial relationships that could be construed as a potential conflict of interest.
