# Supplementary material for: Identification of Quantitative Trait Loci and Water Environmental Interactions for Developmental Behaviors of Leaf Greenness in Wheat
Source: Front Plant Sci. 2016 Mar 8;7:273. doi: 10.3389/fpls.2016.00273 (PMC4782216; doi:10.3389/fpls.2016.00273)
Supplement: Supplementary file 1 [file Table1.DOCX]

**TABLE S1 |Unconditional epistatic effects and interacting effects of epistatic QTL× water environment of identified QTLs for the greenness of flag leaf**

| *QTLi^a^* | Flanking markers | *QTLj^a^* | Flanking markers | Stage*^b^* | *AA^c^* | *AAE^d^* | *H*^2^(*AA*)*^c^*(%) | *H*^2^(*AAE*)*^d^*(%) |
| --- | --- | --- | --- | --- | --- | --- | --- | --- |
| *Qspad.acs-1A.3* | Xgwm357-Xgwm633 | *Qspad.acs-4D.2* | Xksum180-Xwmc48 | S1 | -1.24^***^ | -0.71^***^(E2_DS_), -0.76^***^(E3_DS_), 0.70^***^(E2_WW_),0.73^***^(E3_WW_) | 1.91 | 1.04 |
| *Qspad.acs-1A.4* | Xgwm33-Xwmc818 | *Qspad.acs-5D.4* | Xgwm232-Xcfd78 | S1 | -1.16^***^ | -0.79^***^(E1_DS_), 0.81^***^(E1_WW_), 0.85^***^(E3_WW_) | 1.83 | 1.26 |
|  |  |  |  | S2 | -1.02^***^ | -0.77^***^(E1_DS_), -0.80^***^(E2_DS_), 0.80^***^(E3_WW_) | 1.78 | 1.14 |
|  |  |  |  | S3 | -0.97^***^ | -0.84^***^(E1_DS_), 0.81^***^(E1_WW_), 0.84^***^(E3_WW_) | 1.75 | 1.41 |
| *Qspad.acs-1A.5* | Xgwm497-Xpsp3003 | *Qspad.acs-7B.4* | Xbarc72-Xgwm43 | S1 | -1.29^***^ |  | 1.95 |  |
|  |  |  |  | S2 | -1.18^***^ | 0.66^***^(E1_DS_) | 2.20 | 0.88 |
|  |  |  |  | S3 | -0.93^***^ | -0.89^***^(E2_DS_), -0.83^***^(E3_DS_), 0.89^***^(E2_WW_) | 1.41 | 1.44 |
| *Qspad.acs-1A.6* | Xgwm164-Xcfd59 | *Qspad.acs-4D.3* | Xmag1163-Xwmc399 | S2 | -1.04^***^ |  | 1.56 |  |
| *Qspad.acs-1A.7* | Xksum41-Xwmc254 | *Qspad.acs-3D.2* | Xgwm314-Xgwm456 | S3 | -1.04^***^ | -0.69^***^(E1_DS_) | 1.61 | 0.84 |
|  |  |  |  | S5 | -0.42^***^ | -0.33^**^(E1_DS_), -0.35^***^(E3_DS_) | 1.29 | 2.29 |
| *Qspad.acs-1D* | Xwmc216-Xgwm642 | *Qspad.acs-2D.4* | Xwmc112-Xgwm484 | S2 | -1.06^***^ | -0.64^***^(E1_DS_), 0.66^***^(E2_WW_) | 1.63 | 0.95 |
| *Qspad.acs-2A.7* | Xgwm512-Xgwm30 | *Qspad.acs-4B.4* | Xgwm495-Xgwm251 | S1 | -1.19^***^ |  | 2.22 |  |
|  |  |  |  | S2 | -1.15^***^ |  | 1.96 |  |
| *Qspad.acs-2A.8* | Xgwm515-Xwmc644 | *Qspad.acs-7A.3* | Xgwm233-Xbarc1167 | S1 | -1.16^***^ |  | 1.94 |  |
|  |  |  |  | S2 | -1.12^***^ |  | 2.03 |  |
|  |  |  |  | S3 | -1.01^***^ | -0.53^***^(E3_DS_) | 1.92 | 0.87 |
| *Qspad.acs-2A.9* | Xmag3253-Xwmc522 | *Qspad.acs-3D.2* | Xgwm314-Xgwm456 | S4 | -0.95^***^ | -0.92^***^(E1_DS_), -0.90^***^(E3_DS_), 0.58^***^(E1_WW_), 0.61^***^(E3_WW_) | 1.13 | 2.11 |
| *Qspad.acs-2B.5* | Xgdm87-Xgwm388 | *Qspad.acs-2D.5* | Xcfd50-Xbarc95 | S1 | 1.02^***^ |  | 1.86 |  |
| *Qspad.acs-2B.6* | Xmag3319-Xwmc223 | *Qspad.acs-2D.6* | Xbarc219-Xgwm349 | S2 | 1.01^***^ |  | 1.72 |  |
| *Qspad.acs-2B.7* | Xgwm630-Xksum248 | *Qspad.acs-3A.6* | Xbarc1113-Xwmc153 | S4 | 0.78^***^ | -0.67^***^(E1_DS_), -0.54^***^(E3_DS_), 0.31^**^ (E1_WW_), 0.37^**^(E2_WW_) | 1.24 | 2.38 |
|  |  |  |  | S5 | -0.45^***^ | -0.33^**^(E3_DS_) | 1.08 | 2.54 |
| *Qspad.acs-2D.1* | Xbarc95-Xbarc219 | *Qspad.acs-3B.9* | Xgwm108-Xpsp3035 | S1 | 1.01^***^ | 0.63^***^(E2_WW_) | 1.68 | 0.93 |
|  |  |  |  | S2 | 1.08^***^ |  | 1.83 |  |
| *Qspad.acs-2D.2* | Xgwm608-Xgwm539 | *Qspad.acs-6B.6* | Xgwm273-Xgwm70 | S2 | -1.06^***^ |  | 1.75 |  |
| *Qspad.acs-2D.3* | Xcfd62-Xksum76 | *Qspad.acs-6A.2* | Xbarc171-Xgwm427 | S1 | -1.51^***^ | 0.71^***^(E2_WW_) | 3.29 | 1.11 |
|  |  |  |  | S2 | -1.37^***^ |  | 2.61 |  |
|  |  |  |  | S3 | -0.97^***^ | -0.65^***^(E1_DS_) | 1.64 | 0.89 |
| *Qspad.acs-3A.1* | Xwmc11-Xgwm391 | *Qspad.acs-5D.4* | Xgwm232-Xcfd78 | S1 | 1.19^***^ |  | 2.22 |  |
| *Qspad.acs-3A.4* | Xwmc505-Xwmc343 | *Qspad.acs-6B.6* | Xgwm273-Xgwm70 | S1 | 1.10^***^ | -0.67^***^(E3_DS_), 0.63^***^(E2_WW_) | 2.03 | 0.89 |
| *Qspad.acs-3A.5* | Xcfd193-Xcfa2234 | *Qspad.acs-3D.1* | Xwmc529-Xwmc631 | S1 | 1.16^***^ | 0.61^***^(E3_WW_) | 1.95 | 0.86 |
| *Qspad.acs-3A.6* | Xbarc1113-Xwmc153 | *Qspad.acs-5D.5* | Xwmc212-Xgwm292 | S2 | 1.21^***^ |  | 2.24 |  |
| *Qspad.acs-3B.4* | Xwmc51-Xpsp3144 | *Qspad.acs-7A.3* | Xgwm233-Xbarc1167 | S1 | 0.99^***^ |  | 1.62 |  |
| *Qspad.acs-3B.5* | Xgdm64-Xwmc51 | *Qspad.acs-3D.2* | Xgwm314-Xgwm456 | S1 | 0.97^***^ | 0.70^***^(E1_WW_) | 1.70 | 0.90 |
| *Qspad.acs-3B.6* | Xwmc510-Xgwm181 | *Qspad.acs-4D.3* | Xmag1163-Xwmc399 | S1 | -1.04^***^ |  | 1.90 |  |
| *Qspad.acs-3B.7* | Xgwm299-Xksum45 | *Qspad.acs-6B.6* | Xgwm273-Xgwm70 | S1 | 1.07^***^ |  | 2.03 |  |
|  |  |  |  | S2 | 0.96^***^ |  | 1.88 |  |
| *Qspad.acs-3B.8* | Xwmc231-Xbarc173 | *Qspad.acs-7B.5* | Xbarc65-Xbarc72 | S3 | -0.91^***^ | -0.95^***^(E1_DS_), -0.90^***^(E3_DS_), 0.92^***^(E1_WW_), 0.94^***^(E3_WW_) | 1.71 | 0.88 |
|  |  |  |  | S4 | -0.54^***^ | -0.88^***^(E1_DS_), 0.42^***^(E3_WW_) | 1.08 | 2.39 |
| *Qspad.acs-3D.1* | Xwmc529-Xwmc631 | *Qspad.acs-6A.3* | Xgwm169-Xwmc580 | S4 | -1.07^***^ | -0.93^***^(E1_DS_), 0.50^***^(E3_WW_) | 1.40 | 1.49 |
| *Qspad.acs-3D.2* | Xgwm314-Xgwm456 | *Qspad.acs-6A.5* | Xwmc553-Xwmc179 | S1 | 1.32^***^ |  | 2.60 |  |
|  |  |  |  | S2 | 1.23^***^ |  | 2.09 |  |
|  |  |  |  | S3 | 1.00^***^ |  | 1.76 |  |
| *Qspad.acs-4A.2* | Xmag3733-Xwmc707 | *Qspad.acs-4A.3* | Xksum51-Xgwm637 | S4 | -1.22^***^ | -0.86^***^(E1_DS_), -0.83^***^(E3_DS_), 0.50^***^(E1_WW_), 0.62^***^(E3_WW_) | 1.24 | 1.61 |
| *Qspad.acs-4B.4* | Xgwm495-Xgwm251 | *Qspad.acs-4D.1* | Xwmc457-Xgdm125 | S1 | -1.05^***^ |  | 1.85 |  |
|  |  |  |  | S3 | -0.89^***^ | -0.55^***^(E2_DS_), 0.56^***^(E2_WW_), 0.63^***^(E3_WW_) | 1.59 | 1.03 |
| *Qspad.acs-4D.1* | Xwmc457-Xgdm125 | *Qspad.acs-7A.4* | Xgwm471-Xgwm60 | S1 | 0.99^***^ |  | 1.71 |  |
| *Qspad.acs-5B.5* | Xbarc232-Xgdm116 | *Qspad.acs-7B.6* | Xbarc1073-Xgwm68 | S1 | -1.26^***^ | -0.54^***^(E1_DS_), 0.53^***^(E3_WW_) | 2.45 | 0.99 |
|  |  |  |  | S2 | -1.26^***^ |  | 2.25 |  |
| *Qspad.acs-5B.6* | Xwmc376-Xgwm335 | *Qspad.acs-5B.7* | Xwmc740-Xbarc308 | S1 | -1.32^***^ |  | 1.48 |  |
| *Qspad.acs-5D.2* | Xbarc205-Xgwm232 | *Qspad.acs-7A.5* | Xgwm260-Xwmc422 | S2 | -1.06^***^ | -0.59^***^(E1_DS_), 0.58^***^(E2_WW_) | 1.75 | 1.06 |
| *Qspad.acs-5D.3* | Xgwm174-Xgdm138 | *Qspad.acs-6A.4* | Xbarc113-Xwmc621 | S1 | 1.34^***^ |  | 2.09 |  |
|  |  |  |  | S2 | -0.93^***^ | -0.84^***^(E2_DS_), -0.83^***^(E3_DS_),0.82^***^(E3_WW_) | 1.65 | 1.54 |
|  |  |  |  | S3 | -0.75^***^ | -0.51^***^(E2_DS_), -0.64^***^(E3_DS_), 0.56^***^(E3_WW_) | 1.25 | 1.59 |
| *Qspad.acs-6A.1* | Xwmc201-Xwmc684 | *Qspad.acs-7B.5* | Xbarc65-Xbarc72 | S2 | -1.15^***^ | 0.53^***^(E2_WW_) | 2.33 | 0.87 |
|  |  |  |  | S3 | -0.82^***^ | -0.60^***^(E1_DS_), -0.56^***^(E3_DS_), 0.54^***^(E3_WW_) | 1.48 | 1.70 |
| *Qspad.acs-6B.2* | Xcfd13-Xwmc737 | *Qspad.acs-7B.7* | Xwmc426-Xbarc65 | S2 | -1.17^***^ |  | 2.21 |  |
|  |  |  |  | S3 | -0.89^***^ | 0.52^***^(E1_DS_) | 1.58 | 0.82 |
| *Qspad.acs-6B.4* | Xgwm644-Xmag1266 | *Qspad.acs-7B.7* | Xwmc426-Xbarc65 | S1 | -1.02^***^ | 0.60^***^(E2_WW_), 0.66^***^(E3_WW_) | 1.83 | 1.06 |
| *Qspad.acs-6B.5* | Xwmc494-Xgwm508 | *Qspad.acs-6B.1* | Xgwm508-Xmag1378 | S1 | -1.45^***^ |  | 3.11 |  |
|  |  |  |  | S2 | -1.18^***^ |  | 2.10 |  |
| *Qspad.acs-6D* | Xgwm582-Xwmc469 | *Qspad.acs-7D.4* | Xbarc53-Xgwm37 | S1 | 1.12^***^ |  | 2.10 |  |
|  |  |  |  | S2 | 1.08^***^ |  | 2.15 |  |

|  |  |  |  | S3 | 0.82^***^ | -0.89^***^(E1_DS_), -0.91^***^(E3_DS_), 0.83^***^(E1_WW_), 0.80^***^(E2_WW_) | 1.82 | 2.34 |
| --- | --- | --- | --- | --- | --- | --- | --- | --- |

*^a^* QTL*_i_* and QTL*_j_* are a pair of QTL detected by two-dimensional searching. *^b^* S1 to S5 are as shown in Table 1. *^c^AA*, the direction of the epistatic effect: a positive value means that the parent-type effect is greater than the recombinant-type effect, and the negative value means that the parent-type effect is less than the recombinant-type effect; *^*^P*≤0.01,^**^*P*≤0.005 and ^***^*P*≤0.001; *H*^2^(*AA*) (%) indicates the phenotypic variance explained by epistatic QTL. *^d^AAE*, the interaction effect of epistatic QTL×environment in drought stress (DS) and the well-watered (WW) conditions in E1 to E3 shown in table 1. *H*^2^(*AAE*) (%) indicates the phenotypic variance explained by an epistatic QTL×environment interaction.
